# Prediction Model of New Onset Atrial Fibrillation in Patients with Acute Coronary Syndrome

**DOI:** 10.1155/2023/3473603

**Published:** 2023-02-23

**Authors:** Na Wu, Junzheng Li, Xiang Xu, Zhiquan Yuan, Lili Yang, Yanxiu Chen, Tingting Xia, Qin Hu, Zheng Chen, Chengying Li, Ying Xiang, Zhihui Zhang, Li Zhong, Yafei Li

**Affiliations:** ^1^Department of Epidemiology, College of Preventive Medicine, Army Medical University, Chongqing 400038, China; ^2^Evidence-based Medicine and Clinical Epidemiology Center, Army Medical University, Chongqing 400038, China; ^3^Department of Cardiology and the Center for Circadian Metabolism and Cardiovascular Disease, Southwest Hospital, Army Medical University, Chongqing 400038, China; ^4^Department of Information, Xinqiao Hospital, Army Medical University, Chongqing 400038, China; ^5^Cardiovascular Disease Center, Third Affiliated Hospital of Chongqing Medical University, Chongqing 401120, China

## Abstract

**Objective:**

Atrial fibrillation (AF) is one of the most common complications of acute coronary syndrome (ACS) patients. Possible risk factors related to new-onset AF (NOAF) in ACS patients have been reported in some studies, and several prediction models have been established. However, the predictive power of these models was modest and lacked independent validation. The aim of this study is to define risk factors of NOAF in patients with ACS during hospitalization and to develop a prediction model and nomogram for individual risk prediction.

**Methods:**

Retrospective cohort studies were conducted. A total of 1535 eligible ACS patients from one hospital were recruited for model development. External validation was performed using an external cohort of 1635 ACS patients from another hospital. The prediction model was created using multivariable logistic regression and validated in an external cohort. The discrimination, calibration, and clinical utility of the model were evaluated, and a nomogram was constructed. A subgroup analysis was performed for unstable angina (UA) patients.

**Results:**

During hospitalization, the incidence of NOAF was 8.21% and 6.12% in the training and validation cohorts, respectively. Age, admission heart rate, left atrial diameter, right atrial diameter, heart failure, brain natriuretic peptide (BNP) level, less statin use, and no percutaneous coronary intervention (PCI) were independent predictors of NOAF. The AUC was 0.891 (95% CI: 0.863–0.920) and 0.839 (95% CI: 0.796–0.883) for the training and validation cohort, respectively, and the model passed the calibration test (*P* > 0.05). The clinical utility evaluation shows that the model has a clinical net benefit within a certain range of the threshold probability.

**Conclusion:**

A model with strong predictive power was constructed for predicting the risk of NOAF in patients with ACS during hospitalization. It might help with the identification of ACS patients at risk and early intervention of NOAF during hospitalization.

## 1. Introduction

Acute coronary syndrome (ACS) is a life-threatening heart condition associated with a sudden reduction in blood flow to the heart [1]. ACS includes ST-elevation myocardial infarction (STEMI), non-ST elevation myocardial infarction (NSTEMI), and unstable angina (UA) [2]. In the Asia-Pacific region, the mortality rate during hospitalization is over 5% [3]. AF is one of the most common complications of ACS patients. The incidence of new-onset AF (NOAF) ranged from 2.4% to 37% in ACS patients during hospitalization [4–16]. NOAF during hospitalization significantly increases the risk of a major adverse cardiac event, stroke, systemic embolism, and mortality [9, 10, 14, 17, 18]. Therefore, NOAF is an important health problem for patients with ACS during hospitalization.

Early detection and intervention in NOAF among ACS patients during hospitalization are particularly important. As a result, many efforts have been made previously. Possible risk factors related to NOAF in ACS patients have been reported in some studies, and several prediction models have been established to identify high-risk individuals [13, 19–21]. However, these models had only modest predictive value with the c-statistics ranging from 0.62 to 0.79, and were not comprehensively evaluated or external validated. Owing to the lack of a specific and practical predictive method, the development of a predictive model that has satisfactory predictive value based on clinical characteristics at admission becomes desirable. In addition, ACS includes three subtypes, of which UA is the most common one [2]. The risk factors for NOAF in different subtypes of ACS may vary, and the prediction model for NOAF in UA patients is rare.

We therefore conducted this study to (1) define risk factors for NOAF in patients with ACS during hospitalization, (2) develop a prediction model and nomogram for NOAF risk in ACS patients and comprehensively evaluate and externally validate the model, and (3) perform subgroup analysis to develop a prediction model for NOAF risk in UA patients specifically.

## 2. Methods

### 2.1. Study Participants

Retrospective cohort studies were conducted. We retrospectively enrolled 5403 consecutive ACS patients admitted to the Department of Cardiology, The First Affiliated Hospital of the Army Medical University (Third Military Medical University) in Chongqing, China, from January 2010 to December 2019 for the training cohort. In addition, we retrospectively enrolled 2316 consecutive ACS patients admitted to the Department of Cardiology, Second Affiliated Hospital of the Army Medical University, from January 2017 to December 2019 for external validation cohort. Patients who were at least 18 years old and diagnosed with ACS were eligible for this study. Patients with a history or record of AF at admission, valvular diseases, infections, malignant tumors, systemic inflammatory diseases, incomplete records of echocardiography, admission heart rate, circulating brain natriuretic peptide (BNP), and other important data were excluded from the study. ACS was defined according to the guideline [22]. Continuous ECG monitoring was routinely carried out for all ACS patients throughout the period of hospitalization so that asymptomatic AF could also be detected and diagnosed. AF was defined according to the 2016 ESC Guidelines [23]. Finally, 1535 and 1635 ACS patients were eligible for the training and validation cohort, respectively. The study was approved by the Ethics Committee of the Southwest Hospital of the Army Medical University. The research was conducted in accordance with the Helsinki declaration guidelines and all procedures listed here were carried out in compliance with the approved guidelines. Because this is a retrospective study and all the parameters assessed were routinely obtained in the hospitals so that no additional investigations or procedures were carried out, informed consent was waived by the Ethics Committee of Southwest Hospital of the Army Medical University.

### 2.2. Data Collection

We collected information on demographics (age and gender) and comorbidities (hypertension, diabetes, cardiomyopathy, cerebral infarction, chronic obstructive pulmonary disease, and chronic renal insufficiency). In addition, data from physical examination (height, weight, heart rate, and blood pressure) and Killip classification at admission and the first biochemical/echocardiography examination (blood routine, liver and kidney function, cardiac troponin, circulating high-sensitivity C-reactive protein, circulating BNP, etc.) were collected. The information about medication and percutaneous coronary intervention (PCI) received during hospitalization was also collected from the electronic medical records. NOAF is defined as the onset of AF during hospitalization, and there is no history or record of AF before.

### 2.3. Statistical Analysis

The descriptive statistics are presented as frequency counts and proportions for categorical data, means and standard deviation (SD) for continuous variables that were normally distributed, and medians and interquartile ranges (25th–75th percentile) for continuous variables that were not normally distributed. To test the differences in means and proportions between two groups, we used a *t*-test and a chi-square test, respectively. Multiple imputation was used for the missing BMI, neutrophil count, and leukocyte count (which accounted for <10% of observations). Previous studies found that ACS patients with the admission heart rate above 80 bpm are at the highest risk of in-hospital mortality [24], suggesting that an admission heart rate above 80 bpm is of great significance for ACS patients. In the training cohort, the median admission heart rate of NOAF patients was over 85 bpm, and the median admission heart rate of patients without NOAF was less than 80 bpm. Therefore, heart rate at admission was transformed into dichotomous variable at 85 bpm. Killip classification at admission was transformed into a dichotomous variable as “heart failure” (Killip classification was II, III, or IV) and “no heart failure” (Killip classification was I). The level of circulating BNP was log-transformed due to a heavy skew in the distribution.

To investigate the risk factors of NOAF, the significance of each variable in the training cohort was assessed by univariate logistic regression analysis. All variables associated with NOAF at a significant level were candidates for multivariable analysis. For the selection of prediction model, according to the TRIPOD statement, logistic regression is used for short-term (for example, 30-day mortality) prognostic outcomes, so multivariable logistic regression is used. Backward elimination was adopted for selection of variables entering the final multivariable logistic regression model. The variance inflation factor (VIF) was used to identify collinearity among the covariates. The collinearity was negligible because the VIFs of the variables were less than 5. The model was validated in the external cohort. The discrimination of the models was assessed by the ROC curve and area under the curve (AUC). The calibration curve was drawn to evaluate the model's calibration degree. The clinical utility of the model was evaluated by decision curve analysis. The nomogram was depicted based on the prediction model.

A two-sided *P* value <0.05 was considered to be statistically significant. All these statistical analyses were conducted using R 3.6.3 (R Foundation for Statistical Computing, Vienna, Austria).

## 3. Results

During the study period, 1535 and 1635 ACS patients were recruited in the training and validation cohorts, respectively. The incidence of NOAF during hospitalization was 8.21% in the training cohort and 6.12% in the validation cohort. The UA, STEMI, and NSTEMI patients were 820 (53.42%), 248 (16.16%), and 467 (30.42%) in the training cohort, and 1288 (78.78%), 128 (7.83%), and 219 (13.39%) in the validation cohort. The median length of hospital stay was 6 days and 7 days in the training and validation cohorts, respectively.

The clinical and demographic characteristics of the patients are presented in Table 1. Compared with patients who did not develop AF, NOAF patients were significantly older, more likely to combine with heart failure, have a higher heart rate at admission, and have higher levels of circulating BNP, D-dimer, fibrinogen, serum creatinine, left atrial diameter, and right atrial diameter. NOAF patients had significantly lower levels of admission systolic blood pressure, lymphocytes, platelets, hemoglobin, triglycerides, albumin, and glomerular filtration rate and were less likely to receive aspirin and PCI during hospitalization in both training and validation cohorts. In addition, there was no significant difference in the ACS subtype between non-NOAF and NOAF patients in the training and validation cohort. The incidence of NOAF for UA, NSTEMI, and STEMI patients were 7.44%, 8.87%, and 9.21%, respectively, in the training cohort and were 5.98%, 6.25%, and 6.85%, respectively, in the validation cohort. It indicated that NOAF was more frequent in those with STEMI than in those with NSTEMI or UA, although the differences were not significant. The variables with significant associations assessed by the univariate logistic regression are shown in Supplementary Table S1. In the final multivariable logistic regression model, the age (Odds ratio (OR): 1.058, 95% confidence interval (CI): 1.036–1.082), admission heart rate ≥85 bpm (OR: 2.207, 95% CI: 1.406–3.654), left atrial diameter (OR: 1.102, 95% CI: 1.054–1.153), right atrial diameter (OR: 1.102, 95% CI: 1.054–1.152), heart failure (OR: 2.663, 95% CI: 1.637–4.331), BNP level (OR: 1.472, 95% CI: 1.013–2.140), use of statins (OR: 0.478, 95% CI: 0.281–0.811), and PCI (OR: 0.504, 95% CI: 0.312–0.814) were independently associated with NOAF (Table 2).

**Figure 1:**
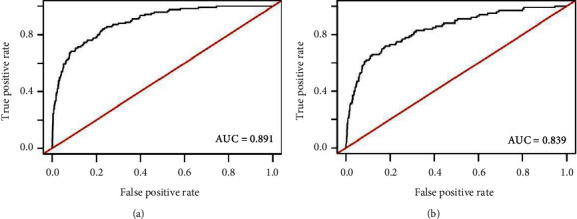
Receiver operating characteristic (ROC) curve for models in predicting NOAF in ACS patients. (a) ROC curve for the model in the training cohort; (b) the ROC curve for the model in the validation cohort. AUC, area under the curve.

**Figure 2:**
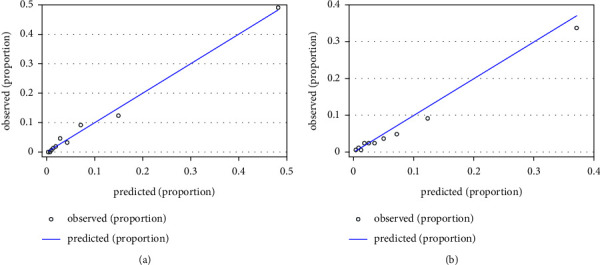
The Hosmer-–Lemeshow calibration curve for the model predicting probability of NOAF in ACS patients. (a) Calibration curve for the model in the training cohort; (b) the calibration curve for the model in the validation cohort. *X*-axis is predicted probability by model and *y*-axis is actual probability of NOAF.

**Figure 3:**
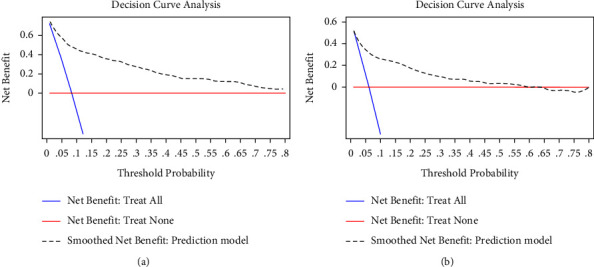
The decision curve analysis (DCA) for the model predicting probability of NOAF in ACS patients. (a) DCA for the model in the training cohort; (b) the DCA for the model in the validation cohort. The decision curve of the prediction model is composed of an *X*-axis which represents continuum of potential thresholds for NOAF risk and a *Y*-axis which represents the net benefit which is obtained by dividing the net true positives by the sample size. The blue curve “net benefit: treat all” shows the net benefit if all ACS patients were intervened for NOAF. The red line “net benefit: treat none” shows the net benefit if no ACS patients were intervened for NOAF. The black dotted line “prediction model” curve shows the net benefit if it is used to select patients for NOAF intervention. For example, if the personal threshold probability of a patient was 40%, the net benefit would be 0.2 when using the prediction model to decide whether to intervene NOAF, which means that there are 20 net detected NOAF per 100 patients.

**Figure 4:**
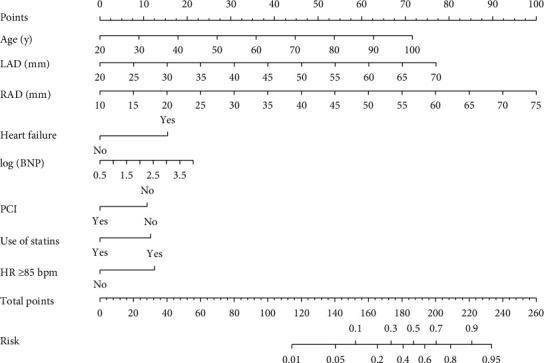
Nomogram to predict NOAF risk during hospitalization in ACS patients. To use the nomogram, an individual patient's value was located on each variable axis, and a line was drawn upward to determine the number of points received for each variable value. The sum of these numbers was located on the total points axis, and a line was drawn downward to the risk axis to determine the risk of NOAF presence. LAD, left atrial diameter; RAD, right atrial diameter; BNP, brain natriuretic peptide; PCI, percutaneous coronary intervention; HR, heart rate.

The ROC curve analysis was used to investigate the discrimination of the prediction model. The AUC was 0.891 (95% CI: 0.863–0.920) and 0.839 (95% CI: 0.796–0.883) in the training and validation cohorts, respectively (Figure 1). The Hosmer and Lemeshow test of the final model showed an effective goodness-of-fit (*P* > 0.05 in both cohorts). The calibration plots presented a good agreement between the predicted probability and actual probability of NOAF (Figure 2). The final decision curve showed that for a threshold probability between 5% and 50%, the model had a positive net benefit (Figure 3). A nomogram was constructed based on the model (Figure 4).

We then performed a subgroup analysis to develop a prediction model for NOAF risk in UA patients specifically. The final multivariable logistic regression model included six predictors that were also predictors of ACS, namely age, heart failure, left atrial diameter, right atrial diameter, BNP level, and no PCI (Table 3). The AUC was 0.894 (95% CI: 0.854–0.934) and 0.844 (95% CI: 0.796–0.891) in training and validation cohorts, respectively (Supplementary Figure S1), and the model passed the calibration test (*P* > 0.05) (Supplementary Figure S2). The clinical utility evaluation shows that the model has a clinical net benefit within a certain range of the threshold probability (5%–50%) (Supplementary Figure S3). A nomogram was also constructed (Supplementary Figure S4).

## 4. Discussion

In this study, we found age, admission heart rate, left atrial diameter, right atrial diameter, heart failure, BNP level, less statin use, and no PCI were independent predictors of NOAF during hospitalization in ACS patients. The prediction model based on these eight variables had good discrimination, calibration, and clinical utility.

The incidence of NOAF during hospitalization in Chinese ACS patients ranged from 6.7% to 13.4%. [13, 16, 25, 26] The incidence variation may be due to different study design, participants, and treatments. In this study, the incidence was 8.21% in the training cohort and 6.12% in the validation cohort, which were similar to those in the previous studies. Early detection of high-risk patients is crucial for the intervention of NOAF.

Several studies had tried to establish prediction models for NOAF in ACS patients. Mazzone and colleagues had established a model to predict NOAF during hospitalization in STEMI patients who underwent PCI, and the model included age, leukocyte count, BNP, and obesity. The C-statistics were 0.734 and 0.76 in the training and validation cohorts, respectively [20]. Two studies reported the plasma BNP level in patients with STEMI is a predictor of NOAF, and the AUC was 0.623 and 0.647, respectively [13, 21]. Yildirim et al. developed a Value of Syntax Score II to predict NOAF in NSTEMI patients who underwent PCI, and the AUC of the model was 0.799 [19]. However, these models had only modest discriminatory power and were not validated or evaluated in terms of their calibration or clinical utility.

In this study, our multivariable analysis revealed several predictors of NOAF. By combining these predictors, we constructed a prediction model. Interestingly, the newly constructed model demonstrated a strong discriminatory performance (with AUC over 0.8) to identify patients with an increased risk of NOAF. This model was externally validated and comprehensively evaluated. In addition to enhancing clinical risk prediction, simplicity, practicability, and costs of predictors should be considered. The model included eight predictors which were more than those in previous studies, but these physiological and examination parameters were routinely obtained in the clinical setting.

The predictors of NOAF were age, admission heart rate, left atrial diameter, right atrial diameter, heart failure, BNP level, less statins use, and no PCI according to our results. It is known that age is an independent risk factor of AF. The incidence of AF approximately doubled for every 10-year increment in age [27]. Previous studies demonstrated that age was an independent risk factor for NOAF in ACS patients [15, 20, 28]. According to a study based on 58 European hospitals, the heart rate at admission is a predictor of in-hospital mortality in patients with ACS [24]. Two studies showed that the NOAF patients had a higher heart rate at admission than non-NOAF [4, 10]. ACS is associated with a sudden reduction in blood flow to the heart. Atrial ischemia causes strong conduction slowing in the ischemic zone, which may stabilize atrial reentry that maintains AF [29]. Heart rate is the most direct indicator of heart activity, and an increased heart rate may reflect a subtle change in cardiac electrophysiology. Left/right atrial diameter is an independent predictor for NOAF occurrence according to some previous studies [9, 26], and increased left/right atrial diameter is a marker of progressive dilatation and remodeling of the left atrial myocardium, which acts as a substrate for AF initiation and maintenance. In addition, heart failure is a risk factor for NOAF in our study whereas it is rarely reported in previous studies as a risk factor for NOAF during hospitalization. But it is known that heart failure and AF coincide in many patients. Heart failure and AF can cause and exacerbate each other through mechanisms such as structural cardiac remodeling, activation of neurohormonal mechanisms, and rate-related impairment of left ventricular function [30]. BNP is a neurohormone released from ventricular myocytes in response to acute volume and/or pressure overload is associated with the severity of left ventricular dysfunction, impaired hemodynamic parameters, and increased left ventricular end-diastolic pressure. Consistent with previous studies [26, 31, 32], we found that an increased circulating BNP level was a risk factor for NOAF in ACS patients.

The use of statins is a protective factor for NOAF according to the results. Several meta-analyses have been published, demonstrating that the use of statins was significantly associated with an AF reduction around 30% in patients who experienced cardiac surgery [33–35] or electrical cardioversion [36], patients with coronary artery disease [37], or ACS [38]. Accumulated evidence has indicated that inflammation characterized by an elevated level of CRP and IL-6 may play important role in AF occurrences [39]. Statins have an established anti-inflammatory effect by inhibiting IL-6, tumor necrosis factor *α* (TNF-*α*) production, and nuclear factor kappa B (NF-*κ*B) activation, and suppressing initiation and progression of AF [38]. PCI alters the natural history of ACS, and it is a protective factor for NOAF in this study, which is consistent with a previous study which reported PCI could reduce the risk of developing NOAF in patients with acute myocardial infarction [25].

In the training cohort, the NOAF patients were more likely to have combined with cardiomyopathy, but in the validation cohort, there were no significant differences in the proportion of cardiomyopathy between non-NOAF and NOAF groups. Since the training and validation cohorts were selected from a single center, respectively, some characteristics of ACS patients might be different between these two hospitals, and selection bias might be introduced. Even though the proportions of patients combined with cardiomyopathy were different between non-NOAF and NOAF groups in training cohort, after adjusting confounding factors, cardiomyopathy was not a predictor in the final multivariable prediction model. Therefore, the impact of this selection bias on the study results was limited.

UA is the most common subtype of ACS, and it accounted for 46%–91.3% of the total ACS diagnosed [40, 41]. In this study, we found that 66.5% of the ACS patients were UA. The incidences of NOAF during hospitalization ranged from 6.7% to 11.4% for UA patients [13, 19, 42], and NOAF also increased the risk of renal failure, stroke, and mortality. UA patients had different risk factors compared with STEMI/NSTEMI patients [43]. According to previous studies, the incidences and prognoses of NOAF among different subtypes of ACS were also different. NOAF was more frequent in those with STEMI than in those with NSTEMI or UA [17, 44], which was consistent with our results. The incidence of NOAF for UA, NSTEMI and STEMI patients was 7.44%, 8.87% and 9.21%, respectively, in the training cohort, and was 5.98%, 6.25% and 6.85%, respectively, in the validation cohort, although these differences were not significant. NOAF had a larger impact in NSTEMI on the risk of death, stroke, or recurrent myocardial infarction than in STEMI and UA [44, 45]. So we expect the risk factors for NOAF in different subtypes of ACS may vary. But there were few studies aiming to build a prediction model for NOAF in UA patients specifically. In the subgroup analysis of UA patients, we found there were only six predictors in the final model. Except for heart rate at admission and less statin use, the other predictors were the same as those of ACS patients. In all ACS patients, the median heart rate was 78 bpm in the non-NOAF group and 87.5 bpm in the NOAF group. And in UA patients, the median heart rate was also 78 bpm in the non-NOAF group but 82 bpm in the NOAF group. Thus, UA patients had a lower admission heart rate, and the differences in admission heart rate between NOAF and non-NOAF groups were smaller in UA. When included in the multivariable prediction model, admission heart rate was not significant. In all ACS patients, the rate of statins use was 87.4% and 70.6% in the non-NOAF and NOAF groups, respectively. While in the UA patients, the rate of statin use was significantly higher (96.4% and 85.2% in the non-NOAF and NOAF groups, respectively). Moreover, the pathology between UA and STEMI/NSTEMI patients were different. Compared with STEMI/NSTEMI patients, individuals with UA do not experience acute cardiomyocyte injury/necrosis, have a substantially lower risk of death, and appear to derive less benefit from intensified antiplatelet therapy as well as an invasive strategy within 72 h [46]. The driving inflammation process for AF between UA and STEMI/NSTEMI patients might be different. Several studies have investigated the effect of prior statin therapy on NOAF in ACS, but the effect in UA patients was seldom demonstrated. More evidence are needed to elucidate the effect of statin on NOAF among different subtypes of ACS.

Some predictors of NOAF in this model are also identified predictors of ACS such as increased age, size of the atria, and BNP [43, 47–50]. However, the risk factors that this study mainly focuses on are the characteristics of ACS patients at admission, such as Killip classification II-IV at admission, a heart rate over 85 bpm at admission, statins, and PCI during hospitalization. These are specific factors for hospitalized ACS patients and can help clinicians classify high-risk patients accurately in the early stages of hospitalization.

In this study, the rates of ACS patients receiving PCI were 51.53% and 44.04% in the training and validation cohorts, respectively. According to the registration data of coronary intervention in China's mainland, the rate of STEMI patients received PCI increased from 30.72% in 2010 to 67.45% in 2019. Another study enrolled ACS patients from 11 tertiary hospitals in Chengdu which is a city near Chongqing from 2017 to 2019, and the rates of patients received PCI were 80.6%, 50.8%, and 26.9% for STEMI, NSTEMI, and UA patients, respectively [51]. In our study, most of the ACS patients were UA patients, accounting for 53.4% and 78.8% of the training and validation cohorts, respectively. So the rate at which ACS patients received PCI in our study was comparable to that of the whole country and neighboring city. But compared with the percentage of 70%–80% in European and American countries [52], the rate of ACS patients receiving PCI in our study was relatively low. For the medications, the rates of patients receiving statins and aspirins were relatively high in this study, while the rate of patients received ACEI was lower than those of other medications, and even lower than that of the whole country (66.4% in 2011) and neighboring city Chengdu (54.3% in 2017–2019) [51]. It is probably because clinicians were more cautious about prescribing ACEI due to their potential adverse effects on the blood pressure. The treatments of ACS in this study indicated that there were still gaps between the treatment in clinical practice and guidelines. The implementation of guidelines for ACS patients still needs to be further strengthened.

In this study, based on a “real world” retrospective cohort, we identified the most significant predictors for NOAF among all known AF risk factors and then constructed a prediction model with a strong discriminatory performance to identify ACS patients with an increased risk of NOAF during hospitalization. The predictors in this model were routinely examined after admission. They can be easily obtained at the early stage of admission. This model is easy to use in the daily clinical practice and might help clinicians classify high-risk patients accurately in the early stage of hospitalization and improve the intervention of NOAF.

As for the clinical utility of our prediction model, if an ACS patient was predicted to have an increased risk of NOAF during hospitalization, intensive ECG monitoring should be used to detect and diagnose early. Then, preventive therapy might be used for the high-risk individuals. Firstly, statin and ranolazine might reduce the risk of NOAF in ACS patients, according to some studies. An analysis by Bang et al. of ACS patients showed that the absolute risk reduction of NOAF in patients with ACS receiving statin therapy was 5% (10% vs. 15%), with a calculated relative risk reduction (RRR) of 33% [53]. A meta-analysis of statin therapy for prevention of NOAF pooled data from six trials of over 160,000 patients. ACS patients who were taking a statin at baseline had a 35% reduction in NOAF (RR 0.65 (95% CI 0.55 to 0.77)) [38]. In a retrospective analysis of the MERLIN-TIMI36 trial, those ACS patients assigned to ranolazine had a lower incidence of NOAF after one year [54]. Secondly, as for the use of antiarrhythmic agents, there were limited evidences. In a Danish RCT examining those with LV dysfunction and recent MI on dofetilide or placebo, they found that treatment with dofetilide was not associated with a significant reduction in the risk of NOAF [55]. Thirdly, increased risk of thrombo-embolic events in ACS patients with NOAF has been described by several studies. Studies also found reduced mortality in ACS patients with new AF treated with oral anticoagulant (OAC) as compared with treatment without OAC [56, 57]. Despite the clear guideline recommendations for OAC treatment for AF patients, undertreatment remains a serious issue. Some studies found less OAC prescription for new AF as compared with known AF [57, 58]. Possibly, the knowledge that adding OAC to the indicated ACS antiplatelet therapy increases the risk for bleeding leads to withholding the prescription. All in all, the reduction of events with statin, antiarrhythmic agents, and OAC in these specific scenarios has never been well studied in a dedicated randomized controlled trial, and more evidence are needed. We believe with the accumulation of evidence, and after full consideration of the risks and benefits, some preventive therapies might be used for the high-risk patients. And the prediction model provided by our study might be a practical tool in the clinical practice.

## 5. Limitations

Several limitations of this study should be considered when interpreting our results. Firstly, this study was based on retrospective cohorts, and selection bias might be introduced due to collecting data retrospectively. A total of 5403 consecutive ACS patients were recruited in the training cohort, but 3868 of them were excluded, mainly due to the lack of BNP or echocardiography. Secondly, training and validation cohorts were selected from a single center, respectively. Although we validated the prediction model externally and evaluated it comprehensively, more cohorts in other medical centers are needed to further validate it. Thirdly, due to the limitation of retrospective collection of data, we did not have the data of some other previous histories such as heart failure or myocardial infarction, treatment histories before admission, the reason for PCI not performed, and the type of cardiomyopathy in patients. Bias from unmeasured confounding factors may exist. Fourthly, this is an observational study, and observational studies can be used to determine the association rather than ascertain the causal relationship. To ascertain the causal relationship, more prospective clinical studies with long follow-up are needed to support the causality. Finally, we only focus on the NOAF occurrence during hospitalization, and the results of this study need to be validated by well-designed studies with long-termfollow-up.

## 6. Conclusion

In the present study, we demonstrated that age, admission heart rate, left atrial diameter, right atrial diameter, heart failure, BNP level, less statins use, and no PCI were independent predictors of NOAF during hospitalization in ACS patients. A prediction model based on these eight variables was constructed. It might help with the identification of ACS patients at risk and early intervention by NOAF during hospitalization.

## Figures and Tables

**Table 1:** The clinical and demographic characteristics of the ACS patients with/without NOAF.

Characteristics	Training cohort			Validation cohort		
	Non-NOAF*N* = 1409	NOAF *N* = 126	*P* value	Non-NOAF*N* = 1535	NOAF *N* = 100	*P* value
Demographic characteristics						
Age (years)	65.0 (56.0, 73.0)	75.5 (68.0, 80.0)	<0.01	66.0 (57.0, 74.0)	73.0 (65.5, 79.0)	<0.01
Gender (*n* (%))			0.21			0.09
Male	1034 (73.4)	86 (68.3)		1077 (70.2)	62 (62.0)	
Female	375 (26.6)	40 (31.7)		458 (29.8)	38 (38.0)	
BMI (Kg/m^2^)	24.2 (21.8, 27.0)	24.8 (22.0, 27.8)	0.47	24.2 (22.2, 26.5)	24.1 (20.6, 26.2)	0.17
ACS subtypes			0.50			0.88
UA	759 (53.9)	61 (48.4)		1211 (78.9)	77 (77)	
NSTEMI	226 (16.0)	22 (17.5)		120 (7.8)	8 (8)	
STEMI	424 (30.1)	43 (34.1)		204 (13.3)	15 (15)	
Length of stay (days)	6 (5, 8)	7 (5, 1)	<0.01	7 (6, 8)	7 (6, 10)	<0.01
Physical examination at admission						
Heart rate (bpm)	78.0 (70.0, 88.0)	87.5 (74.0, 102.0)	<0.01	75.0 (68.0, 83.0)	78.5 (70.0, 89.5)	<0.01
SBP (mmHg)	130.0 (118.0, 145.0)	125.0 (110.0, 140.0)	0.01	130.0 (117.0, 144.0)	127.0 (114.0, 140.5)	0.10
DBP (mmHg)	78.0 (70.0, 86.0)	74.0 (67.0, 85.0)	0.10	74.0 (66.0, 82.0)	72.0 (63.5, 84.0)	0.26
Killip classification (*n* (%))			<0.01			<0.01
I	885 (62.8)	31 (24.6)		262 (17.1)	7 (7.0)	
II, III, IV	524 (37.2)	95 (75.4)		1273 (82.9)	93 (93.0)	
Comorbidities (*n* (%))						
Hypertension	794 (56.4)	80 (63.5)	0.12	957 (62.3)	59 (59.0)	0.50
Diabetes	475 (33.7)	45 (35.7)	0.65	411 (26.8)	24 (24.0)	0.54
Cardiomyopathy	57 (4.0)	25 (19.8)	<0.01	39 (2.5)	5 (5.0)	0.14
Cerebral infarction	59 (4.2)	9 (7.1)	0.12	146 (9.5)	21 (21.0)	<0.01
COPD	24 (1.7)	6 (4.8)	0.02	62 (4.0)	7 (7.0)	0.15
CRI	36 (2.6)	16 (12.7)	<0.01	57 (3.7)	2 (2.0)	0.37
Medications during hospitalization (*n* (%))						
Statins	1232 (87.4)	89 (70.6)	<0.01	1481 (96.5)	96 (96.0)	0.80
Aspirin	1194 (84.7)	78 (61.9)	<0.01	1360 (88.6)	67 (67.0)	<0.01
Clopidogrel	901 (63.9)	82 (65.1)	0.80	1049 (68.3)	73 (73.0)	0.33
*β*-blocker	893 (63.4)	63 (50.0)	<0.01	1054 (68.7)	77 (77.0)	0.08
ACEI	530 (37.6)	44 (34.9)	0.55	796 (51.9)	68 (68.0)	<0.01
Biochemical examination						
Abnormal cardiac troponin (*n* (%))	650 (46.1)	65 (51.6)	0.24	324 (21.1)	23 (23.0)	0.65
BNP (pg/ml)	80.6 (28.2, 261.0)	432.0 (134.0, 1100.0)	<0.01	46.0 (16.0, 162.0)	179.5 (82.6, 413.0)	<0.01
hsCRP (mg/L)	3.3 (1.4, 10.9)	7.4 (2.0, 12.5)	<0.01	1.9 (0.8, 11.1)	1.4 (0.8, 7.6)	0.14
D-dimer (mg/L)	0.3 (0.2, 0.6)	0.6 (0.3, 1.3)	<0.01	0.2 (0.1, 0.7)	0.4 (0.2, 1.4)	<0.01
Fibrinogen (g/L)	2.8 (2.3, 3.5)	3.0 (2.5, 3.8)	0.02	2.9 (2.4, 3.6)	3.1 (2.6, 4.1)	0.04
WBC (10^9^/L)	7.1 (5.6, 9.0)	6.9 (5.6, 10.2)	0.70	6.3 (5.2, 7.8)	6.9 (5.3, 8.9)	0.03
Neutrophils (10^9^/L)	4.6 (3.4, 6.6)	4.9 (3.5, 8.1)	0.16	4.0 (3.0, 5.3)	4.5 (3.3, 6.6)	<0.01
Lymphocyte (10^9^/L)	1.5 (1.1, 2.0)	1.2 (0.9, 1.7)	<0.01	1.5 (1.2, 1.9)	1.3 (1.0, 1.6)	<0.01
Platelet (10^9^/L)	189.0 (152.0, 231.0)	160.0 (125.0, 205.0)	<0.01	180.0 (145.0, 220.0)	170.0 (126.0, 206.0)	0.02
Hemoglobin (g/L)	135.0 (123.0, 147.0)	131.0 (118.0, 140.0)	<0.01	129.0 (117.0, 141.0)	124.0 (109.5, 138.0)	0.03
TG (mmol/L)	1.3 (1.0, 1.9)	1.0 (0.8, 1.5)	<0.01	1.3 (0.9, 1.8)	1.1 (0.8, 1.4)	<0.01
Total protein (g/L)	64.9 (61.2, 68.1)	64.2 (60.3, 68.0)	0.21	63.9 (60.4, 67.8)	64.3 (58.9, 68.8)	0.70
Albumin (g/L)	38.0 (35.8, 40.2)	35.7 (33.5, 39.2)	<0.01	40.5 (38.1, 42.9)	39.3 (36.5, 42.7)	0.03
GFR (ml·min^−1^·l^−1^)	87.9 (72.7, 97.0)	75.7 (52.5, 89.2)	<0.01	84.1 (68.0, 95.0)	73.3 (53.5, 84.7)	<0.01
Creatinine (*μ*mol/l)	76.0 (65.1, 90.0)	81.4 (68.0, 109.2)	<0.01	78.2 (66.9, 92.1)	85.0 (72.3, 105.7)	<0.01
Echocardiograph*y*						
LAD (mm)	36.0 (33.0, 39.0)	42.0 (38.0, 47.0)	<0.01	34.0 (32.0, 37.0)	41.0 (36.5, 46.0)	<0.01
RAD (mm)	35.0 (32.0, 37.0)	38.5 (35.0, 46.0)	<0.01	35.0 (33.0, 36.0)	38.0 (36.0, 43.0)	<0.01
LVEF (%)	59.0 (53.0, 64.0)	52.5 (42.0, 60.0)	<0.01	63.0 (59.0, 67.0)	62.0 (57.5, 67.0)	0.25
PCI (*n* (%))	758 (53.8)	33 (26.2)	<0.01	694 (45.2)	26 (26.0)	<0.01

Entries are *n* (%) for categorical variables and median (5th percentile–75th percentile) for continuous variables as appropriate. ACS, Acute coronary syndrome; NOAF, new-onset atrial fibrillation; BMI, body mass index; DBP, diastolic blood pressure; SBP, systolic blood pressure; COPD, chronic obstructive pulmonary disease; CRI, chronic renal insufficiency; ACEI, angiotensin-converting enzyme inhibitors; BNP, brain natriuretic peptide; hsCRP, high-sensitivity C-reactive protein; WBC, white blood cell; TG, triglyceride; GFR, glomerular filtration rate; LAD, left atrial diameter; RAD, right atrial diameter; LVEF, left ventricular ejection fraction; PCI, percutaneous coronary intervention.

**Table 2:** Multivariable logistic regression analysis of NOAF in ACS patients.

Variables and intercept	*β*	*β*_*s*_	OR (95% CI)	*P* value
Intercept	−14.673		—	<0.001
Age	0.057	0.368	1.058 (1.036–1.082)	<0.001
Left atrial diameter (mm)	0.098	0.633	1.102 (1.054–1.153)	<0.001
Right atrial diameter (mm)	0.097	0.249	1.102 (1.054–1.152)	<0.001
Heart failure at admission	0.979	0.264	2.663 (1.637–4.331)	<0.001
BNP (log10 transformed)	0.387	0.143	1.472 (1.013–2.140)	0.043
PCI	−0.685	−0.189	0.504 (0.312–0.814)	0.005
Use of statins	−0.739	−0.143	0.478 (0.281–0.811)	0.006
Heart rate at admission (bpm)				
<85			1.000 (reference)	
≥85	0.792	0.205	2.207 (1.406–3.654)	0.001

NOAF, new-onset atrial fibrillation; ACS, acute coronary syndrome; *β*, logistic regression coefficients; *β*s, standardized logistic regression coefficients; OR, odds ratio; CI, confidence interval; BNP, brain natriuretic peptide; PCI, percutaneous coronary intervention.

**Table 3:** Multivariable logistic regression analysis of NOAF in UA patients.

Variables and intercept	*β*	*β*_*s*_	OR (95% CI)	*P* value
Intercept	−14.722		—	<0.001
Age	0.048	0.283	1.049 (1.013–1.087)	0.007
Left atrial diameter (mm)	0.085	0.501	1.089 (1.024–1.158)	0.007
Right atrial diameter (mm)	0.092	0.254	1.097 (1.036–1.161)	0.002
Heart failure at admission	1.388	0.380	4.008 (1.971–8.148)	<0.001
BNP (log10 transformed)	0.750	0.258	2.117(1.206–3.716)	0.009
PCI	−1.372	−0.371	0.254 (0.105–0.613)	0.002

NOAF, new-onset atrial fibrillation, UA, unstable angina; *β*, logistic regression coefficients; *β*s, standardized logistic regression coefficients; OR, odds ratio; CI, confidence interval; BNP, brain natriuretic peptide; PCI, percutaneous coronary intervention.

## Data Availability

The research article data used to support the findings of this study are available from the corresponding author upon request.

## Supplementary Materials

Supplementary Materials Supplemental Table S1. Univariable logistic regression analysis of NOAF in ACS patients. Supplemental Figure S1. Receiver operating characteristic (ROC) curvefor models in predicting NOAF in UA patients. Supplemental Figure S2. The Hosmer-Lemeshow calibration curve for models in predicting NOAF in UA patients. Supplemental Figure S3. The Decision curve analysis (DCA) for the model predicting probability of NOAF in UA patients. Supplemental Figure S4. Nomogram to predict NOAF risk during hospitalization in UA patients.

## References

[1] Libby P. (2013). Mechanisms of acute coronary syndromes and their implications for therapy. *New England Journal of Medicine*.

[2] Timmis A. (2015). Acute coronary syndromes. *BMJ*.

[3] Chan M. Y., Du X., Eccleston D. (2016). Acute coronary syndrome in the Asia-Pacific region. *International Journal of Cardiology*.

[4] Dziewierz A., Siudak Z., Rakowski T., Jakała J., Dubiel J. S., Dudek D. (2010). Prognostic significance of new onset atrial fibrillation in acute coronary syndrome patients treated conservatively. *Cardiology Journal*.

[5] Jons C., Raatikainen P., Gang U. J. (2010). Autonomic dysfunction and new-onset atrial fibrillation in patients with left ventricular systolic dysfunction after acute myocardial infarction: a CARISMA substudy. *Journal of Cardiovascular Electrophysiology*.

[6] Braga C. G., Ramos V., Martins J. (2015). Impact of atrial fibrillation type during acute coronary syndromes: clinical features and prognosis. *Revista Portuguesa de Cardiologia*.

[7] Gonzalez-Pacheco H., Marquez M. F., Arias-Mendoza A. (2015). Clinical features and in-hospital mortality associated with different types of atrial fibrillation in patients with acute coronary syndrome with and without ST elevation. *Journal of Cardiology*.

[8] Ulus T., Isgandarov K., Yilmaz A. S., Vasi I., Moghanchizadeh S. H., Mutlu F. (2018). Predictors of new-onset atrial fibrillation in elderly patients with acute coronary syndrome undergoing percutaneous coronary intervention. *Aging Clinical and Experimental Research*.

[9] Galvao Braga C., Ramos V., Vieira C. (2014). New-onset atrial fibrillation during acute coronary syndromes: predictors and prognosis. *Revista Portuguesa de Cardiologia*.

[10] Almendro-Delia M., Valle-Caballero M. J., Garcia-Rubira J. C. (2014). Prognostic impact of atrial fibrillation in acute coronary syndromes: results from the ARIAM registry. *European Heart Journal: Acute Cardiovascular Care*.

[11] McManus D. D., Huang W., Domakonda K. V. (2012). Trends in atrial fibrillation in patients hospitalized with an acute coronary syndrome. *The American Journal of Medicine*.

[12] Lopes R. D., White J. A., Atar D. (2013). Incidence, treatment, and outcomes of atrial fibrillation complicating non-ST-segment elevation acute coronary syndromes. *International Journal of Cardiology*.

[13] Zhang H., Dong P., Yang X. (2020). Prognostic indicators of new onset atrial fibrillation in patients with acute coronary syndrome. *Clinical Cardiology*.

[14] Nagai M., Itoh T., Ishida M. (2019). New-onset atrial fibrillation in patients with acute coronary syndrome may be associated with worse prognosis and future heart failure. *Journal of Arrhythmia*.

[15] Karatas M. B., Canga Y., Ipek G. (2016). Association of admission serum laboratory parameters with new-onset atrial fibrillation after a primary percutaneous coronary intervention. *Coronary Artery Disease*.

[16] Wang C. L., Chen P. C., Juang H. T., Chang C. J. (2019). Adverse outcomes associated with pre-existing and new-onset atrial fibrillation in patients with acute coronary syndrome: a retrospective cohort study. *Cardiol Ther*.

[17] Lau D. H., Huynh L. T., Chew D. P., Astley C. M., Soman A., Sanders P. (2009). Prognostic impact of types of atrial fibrillation in acute coronary syndromes. *The American Journal of Cardiology*.

[18] Topaz G., Flint N., Steinvil A. (2017). Long term prognosis of atrial fibrillation in ST-elevation myocardial infarction patients undergoing percutaneous coronary intervention. *International Journal of Cardiology*.

[19] Yildirim E., Ermis E., Allahverdiyev S., Ucar H., Cengiz M. (2019). Value of Syntax score II in prediction of new-onset atrial fibrillation in patients with NSTE-ACS undergoing percutaneous coronary intervention. *Angiology*.

[20] Mazzone A., Scalese M., Paradossi U. (2018). Development and validation of a risk stratification score for new-onset atrial fibrillation in STEMI patients undergoing primary percutaneous coronary intervention. *International Journal of Clinical Practice*.

[21] Karabag Y., Rencuzogullari I., Cagdas M. (2018). Association between BNP levels and new-onset atrial fibrillation: a propensity score approach. *Herz*.

[22] Patel M. R., Calhoon J. H., Dehmer G. J. (2017). ACC/AATS/AHA/ASE/ASNC/SCAI/SCCT/STS 2016 appropriate use criteria for coronary revascularization in patients with acute coronary syndromes: a report of the American College of Cardiology appropriate use criteria task force, American association for thoracic surgery, American heart association, American society of echocardiography, American society of nuclear Cardiology, society for cardiovascular angiography and interventions, society of cardiovascular computed tomography, and the society of thoracic surgeons. *Journal of the American College of Cardiology*.

[23] Kirchhof P., Benussi S., Kotecha D. (2016). 2016 ESC Guidelines for the management of atrial fibrillation developed in collaboration with EACTS. *European Heart Journal*.

[24] Jensen M. T., Pereira M., Araujo C. (2014). Heart rate at admission is a predictor of in-hospital mortality in patients with acute coronary syndromes - results from 58 European hospitals - the EURHOBOP study. *European Heart Journal*.

[25] Zhang X., Li G., Zhao Z., Xu Y., Liu T. (2014). The value of CHADS2 score in predicting new-onset atrial fibrillation in Chinese patients with acute myocardial infarction. *International Journal of Cardiology*.

[26] Dorje T., Wang X., Shao M. (2013). Plasma N-terminalpro-brain natriuretic peptide levels predict new-onset atrial fibrillation in patients with acute myocardial infarction. *International Journal of Cardiology*.

[27] Benjamin E. J., Levy D., Vaziri S. M. (1994). Independent risk factors for atrial fibrillation in a population-based cohort. The Framingham Heart Study. *JAMA*.

[28] Shiyovich A., Axelrod M., Gilutz H., Plakht Y. (2019). Early versus late new-onset atrial fibrillation in acute myocardial infarction: differences in clinical characteristics and predictors. *Angiology*.

[29] Sinno H., Derakhchan K., Libersan D., Merhi Y., Leung T. K., Nattel S. (2003). Atrial ischemia promotes atrial fibrillation in dogs. *Circulation*.

[30] Hindricks G., Potpara T., Dagres N. (2020). Corrigendum to: 2020 ESC guidelines for the diagnosis and management of atrial fibrillation developed in collaboration with the European association for cardio-thoracic surgery (EACTS): the task force for the diagnosis and management of atrial fibrillation of the European society of Cardiology (ESC) developed with the special contribution of the European heart rhythm association (EHRA) of the ESC. *European Heart Journal*.

[31] Asanin M., Stankovic S., Mrdovic I. (2012). B-type natriuretic peptide predicts new-onset atrial fibrillation in patients with ST-segment elevation myocardial infarction treated by primary percutaneous coronary intervention. *Peptides*.

[32] Gao X., Zeng R., Liao P., Zhu H., Zhang M. (2016). Relation of N-terminalpro-brain natriuretic peptide and new-onset atrial fibrillation in patients with acute coronary syndrome: a systematic review and meta-analysis. *Scandinavian Journal of Clinical and Laboratory Investigation*.

[33] Kuhn E. W., Liakopoulos O. J., Stange S. (2014). Preoperative statin therapy in cardiac surgery: a meta-analysis of 90,000 patients. *European Journal of Cardio-Thoracic Surgery*.

[34] Fauchier L., Clementy N., Babuty D. (2013). Statin therapy and atrial fibrillation: systematic review and updated meta-analysis of published randomized controlled trials. *Current Opinion in Cardiology*.

[35] Yin L., Wang Z., Wang Y., Ji G., Xu Z. (2010). Effect of statins in preventing postoperative atrial fibrillation following cardiac surgery. *Heart Lung and Circulation*.

[36] Loffredo L., Angelico F., Perri L., Violi F. (2012). Upstream therapy with statin and recurrence of atrial fibrillation after electrical cardioversion. Review of the literature and meta-analysis. *BMC Cardiovascular Disorders*.

[37] Zhou X., Du J. L., Yuan J., Chen Y. Q. (2013). Statin therapy is beneficial for the prevention of atrial fibrillation in patients with coronary artery disease: a meta-analysis. *European Journal of Pharmacology*.

[38] Zhou X., Du J. L., Yuan J., Chen Y. Q. (2013). Statins therapy can reduce the risk of atrial fibrillation in patients with acute coronary syndrome: a meta-analysis. *International Journal of Medical Sciences*.

[39] Wu N., Xu B., Xiang Y. (2013). Association of inflammatory factors with occurrence and recurrence of atrial fibrillation: a meta-analysis. *International Journal of Cardiology*.

[40] Tan H. Q., Liang Y., Zhu J., Liu L. S., Chinese Coordinating Center of Organization to Assess Strategies for Ischemic Syndrome Registry (2002). Clinical characteristics of acute ischemic syndrome in China. *Chinese Medical Journal*.

[41] Gao R., Patel A., Gao W. (2008). Prospective observational study of acute coronary syndromes in China: practice patterns and outcomes. *Heart*.

[42] Tran H. V., Erskine N. A., Nguyen H. L. (2019). Increase in white blood cell count is associated with the development of atrial fibrillation after an acute coronary syndrome. *International Journal of Cardiology*.

[43] Zhang L., Hailati J., Ma X. (2021). Analysis of risk factors for different subtypes of acute coronary syndrome. *Journal of International Medical Research*.

[44] Lopes R. D., Pieper K. S., Horton J. R. (2008). Short- and long-term outcomes following atrial fibrillation in patients with acute coronary syndromes with or without ST-segment elevation. *Heart*.

[45] Gouda P., Savu A., Bainey K. R., Kaul P., Welsh R. C. (2021). Long-term risk of death and recurrent cardiovascular events following acute coronary syndromes. *PLoS One*.

[46] Roffi M., Patrono C., Collet J. P. (2015). ESC guidelines for the management of acute coronary syndromes in patients presenting without persistent ST-segment elevation: task force for the management of acute coronary syndromes in patients presenting without persistent ST-segment elevation of the European society of Cardiology (ESC). *European Heart Journal*.

[47] Talwar S., Squire I. B., Downie P. F., Davies J. E., Ng L. L. (2000). Plasma N terminal pro-brain natriuretic peptide and cardiotrophin 1 are raised in unstable angina. *Heart*.

[48] Sadiq S., Ijaz A., Dawood M. M., Sadiq T. (2022). B-type natriuretic peptide as diagnostic and prognostic marker in various forms of acute coronary syndrome. *Pakistan Journal of Medical Sciences*.

[49] Tokunaga K., Koga M., Yoshimura S. (2020). Left atrial size and ischemic events after ischemic stroke or transient ischemic attack in patients with nonvalvular atrial fibrillation. *Cerebrovascular Diseases*.

[50] Hwang J. H., Park J. B., Kim Y. J. (2017). The prognostic significance of preoperative left ventricular diastolic dysfunction and left atrial enlargement on acute coronary syndrome in kidney transplantation. *Oncotarget*.

[51] Li S. Y., Zhou M. G., Ye T. (2021). Frequency of ST-segment elevation myocardial infarction, non-ST-segment myocardial infarction, and unstable angina: results from a Southwest Chinese Registry. *Reviews in Cardiovascular Medicine*.

[52] Kristensen S. D., Laut K. G., Fajadet J. (2014). Reperfusion therapy for ST elevation acute myocardial infarction 2010/2011: current status in 37 ESC countries. *European Heart Journal*.

[53] Bang C. N., Gislason G. H., Greve A. M., Torp-Pedersen C., Kober L., Wachtell K. (2014). Statins reduce new-onset atrial fibrillation in a first-time myocardial infarction population: a nationwide propensity score-matched study. *European Journal of Preventive Cardiology*.

[54] Scirica B. M., Belardinelli L., Chaitman B. R. (2015). Effect of ranolazine on atrial fibrillation in patients with non-ST elevation acute coronary syndromes: observations from the MERLIN-TIMI 36 trial. *Europace*.

[55] Schmiegelow M. D., Pedersen O. D., Kober L., Seibæk M., Abildstrom S. Z., Torp-Pedersen C. (2011). Incidence of atrial fibrillation in patients with either heart failure or acute myocardial infarction and left ventricular dysfunction: a cohort study. *BMC Cardiovascular Disorders*.

[56] Madsen J. M., Jacobsen M. R., Sabbah M. (2021). Long-term prognostic outcomes and implication of oral anticoagulants in patients with new-onset atrial fibrillation following st-segment elevation myocardial infarction. *American Heart Journal*.

[57] Hofer F., Kazem N., Hammer A. (2021). Long-term prognosis of de novo atrial fibrillation during acute myocardial infarction: the impact of anti-thrombotic treatment strategies. *European Heart Journal - Cardiovascular Pharmacotherapy*.

[58] De Luca L., Di Lenarda A., Rubboli A. (2021). Post-discharge antithrombotic management and clinical outcomes of patients with new-onset or pre-existing atrial fibrillation and acute coronary syndromes undergoing coronary stenting: follow-up data of the MATADOR-PCI study. *European Journal of Internal Medicine*.

