# Supplementary material for: Prediction Model of New Onset Atrial Fibrillation in Patients with Acute Coronary Syndrome
Source: Int J Clin Pract. 2023 Feb 23;2023:3473603. doi: 10.1155/2023/3473603 (PMC9981295; doi:10.1155/2023/3473603)
Supplement: Supplementary Materials — Supplemental Table S1. Univariable logistic regression analysis of NOAF in ACS patients. Supplemental Figure S1. Receiver operating characteristic (ROC) curvefor models in predicting NOAF in UA patients. Supplemental Figure S2. The Hosmer-Lemeshow calibration curve for models in predicting NOAF in UA patients. Supplemental Figure S3. The Decision curve analysis (DCA) for the model predicting probability of NOAF in UA patients. Supplemental Figure S4. Nomogram to predict NOAF risk during hospitalization in UA patients. [file 3473603.f1.DOCX]

**Supplementary Material**

**for**

**"Prediction model of new onset atrial fibrillation in patients with acute coronary syndrome"**

**Supplemental Table S1.** Univariable logistic regression analysis of NOAF in ACS patients.

**Supplemental Figure S1.** Receiver operating characteristic (ROC) curve for models in predicting NOAF in UA patients.

**Supplemental Figure S2.** The Hosmer-Lemeshow calibration curve for models in predicting NOAF in UA patients.

**Supplemental Figure S3.** The Decision curve analysis (DCA) for the model predicting probability of NOAF in UA patients.

**Supplemental Figure S4.** Nomogram to predict NOAF risk during hospitalization in UA patients.

**Supplemental Table S1. Univariable logistic regression analysis of NOAF in ACS patients**

| Variables |  | *OR* (95% *CI*） | *P* value |
| --- | --- | --- | --- |
| Age | | 1.08 (1.06-1.11) | <0.001 |
| Gender (male) | | 0.78 (0.53-1.17) | 0.215 |
| BMI | | 1.02 (0.98-1.06) | 0.395 |
| Heart rate at admission (≥85 bpm) | | 2.69(1.86-3.90) | <0.001 |
| Heart failure at admission | | 5.18 (3.44-7.99) | <0.001 |
| Hypertension | | 1.35 (0.93-1.98) | 0.122 |
| Diabetes | | 1.09 (0.74-1.59) | 0.649 |
| Cardiomyopathy | | 5.87 (3.47-9.70) | <0.001 |
| Cerebral infarction | | 1.76 (0.80-3.47) | 0.127 |
| COPD  .7920 | | 2.89 (1.05-6.76) | 0.023 |
| Chronic renal insufficient | | 5.55 (2.91-10.15) | <0.001 |
| Statins | | 0.35 (0.23-0.53) | <0.001 |
| Aspirin | | 0.29 (0.20-0.43) | <0.001 |
| Clopidogrel | | 1.05 (0.72-1.55) | 0.800 |
| β-blocker | | 0.58 (0.40-0.83) | 0.003 |
| ACEI | | 0.89 (0.60-1.30) | 0.549 |
| Abnormal cardiac troponin | | 1.24 (0.86-1.79) | 0.24 |
| BNP | | 4.37 (3.23-6.00) | <0.001 |
| hsCRP | | 1.08 (1.04-1.12) | <0.001 |
| D-dimer | | 1.04 (1.00-1.09) | 0.058 |
| Fibrinogen | | 1.19 (1.03-1.36) | 0.014 |
| WBC | | 1.03 (0.98-1.09) | 0.206 |
| Neutrophils | | 1.05 (1.00-1.11) | 0.049 |
| Lymphocyte | | 0.51 (0.36-0.71) | <0.001 |
| Platelet | | 0.99 (0.99-1.00) | <0.001 |
| Hemoglobin | | 0.98 (0.98-0.99) | <0.001 |
| TG | | 0.55 (0.40-0.73) | <0.001 |
| Total protein | | 0.98 (0.95-1.01) | 0.232 |
| Albumin | | 0.87 (0.83-0.91) | <0.001 |
| GFR | | 0.97 (0.97-0.98) | <0.001 |
| Creatinine | | 1.00 (0.99-1.01) | 0.07 |
| LAD | | 1.23 (1.19-1.27) | <0.001 |
| RAD | | 1.22 (1.18-1.27) | <0.001 |
| LVEF | | 0.95 (0.94-0.97) | <0.001 |
| PCI | | 0.30 (0.20-0.45) | <0.001 |

ACS, Acute coronary syndrome; NOAF, new-onset atrial fibrillation; BMI, body mass index; COPD, chronic obstructive pulmonary disease; ACEI, angiotensin-converting enzyme inhibitors; BNP, brain natriuretic peptide; hsCRP, high sensitivity C-reactive protein; WBC, white blood cell; TG, triglyceride; GFR, glomerular filtration rate; LAD, left atrial diameter; RAD, right atrial diameter; LVEF, left ventricular ejection fraction; PCI, percutaneous coronary intervention.


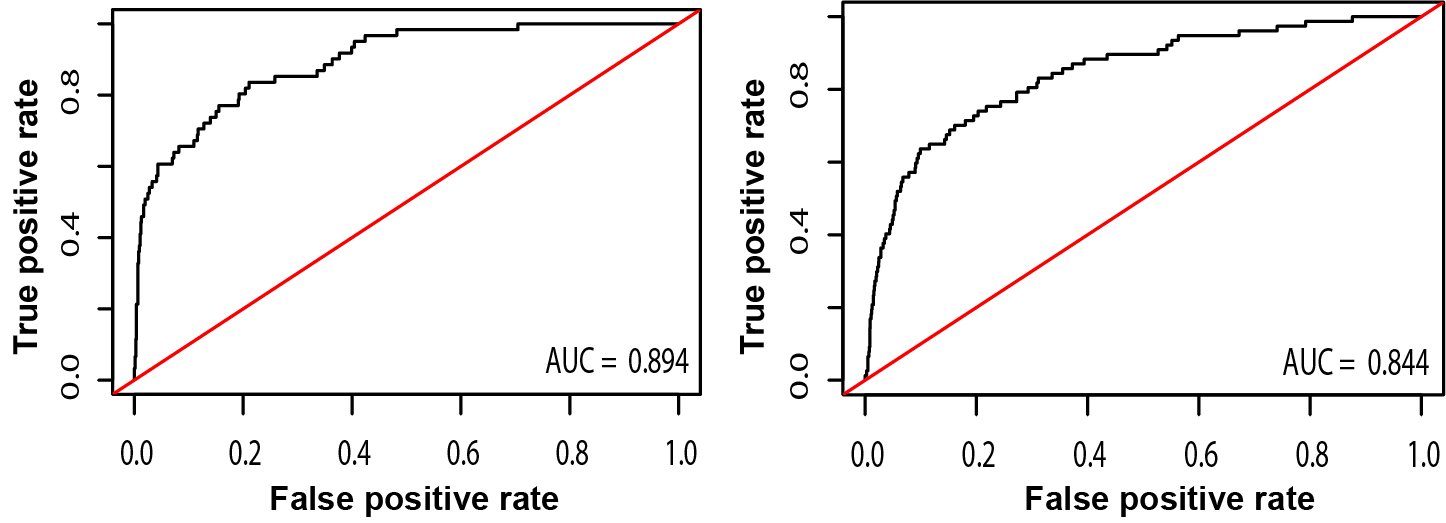


**Supplemental Figure S1.** Receiver operating characteristic (ROC) curve for models in predicting NOAF in UA patients. Left, the ROC curve for the model in the training cohort; right, the ROC curve for the model in the validation cohort. AUC, area under the curve.


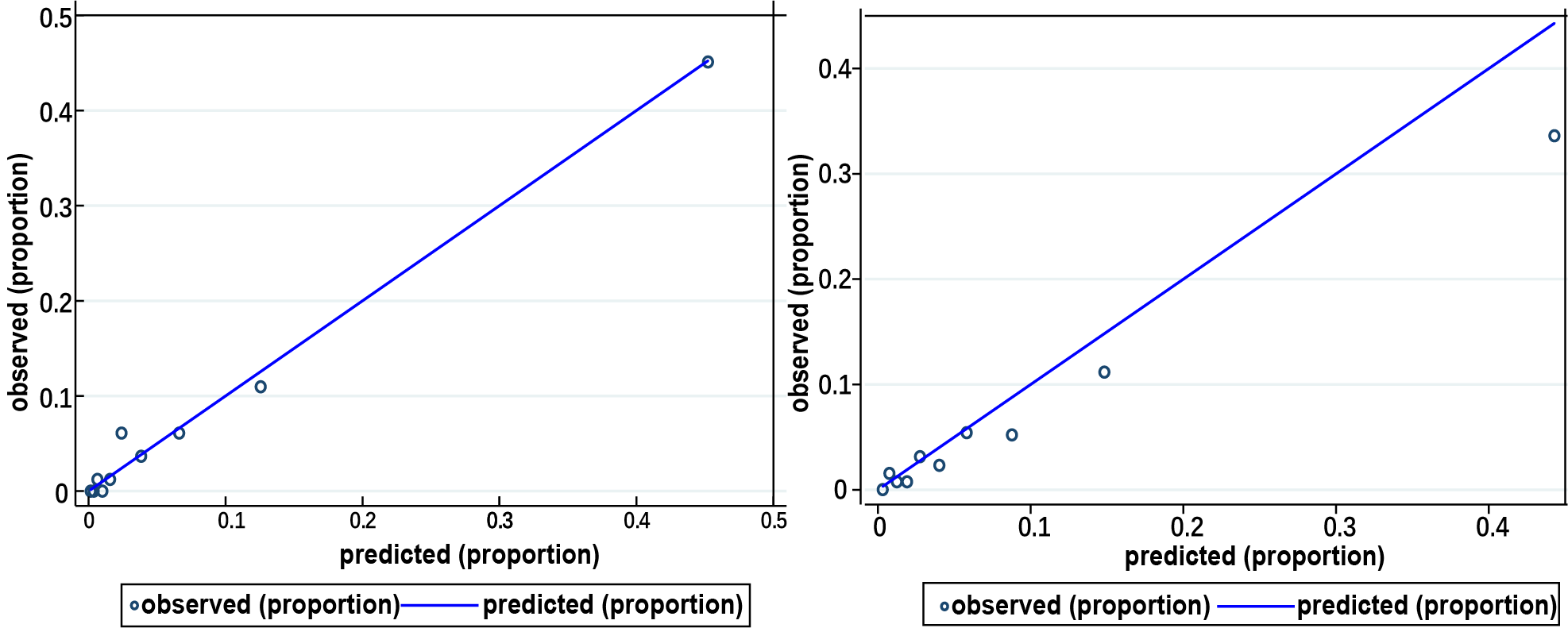


**Supplemental Figure S2.** The Hosmer-Lemeshow calibration curve for models in predicting NOAF in UA patients. Left, the calibration curve for the model in the training cohort; right, the calibration curve for the model in the validation cohort. X-axis is predicted probability by model and y-axis is actual probability of NOAF.


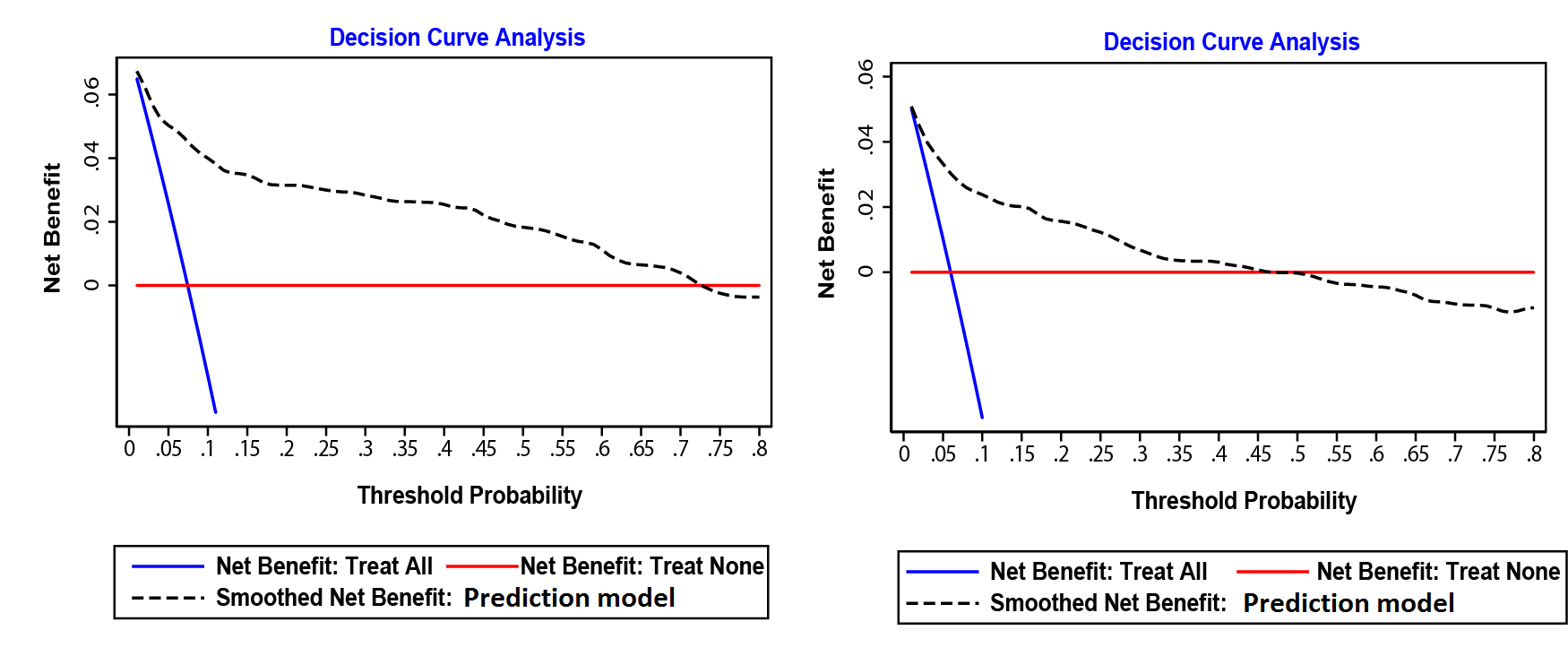


**Supplemental Figure S3.** The Decision curve analysis (DCA) for the model predicting probability of NOAF in UA patients. Left, the DCA for the model in the training cohort; right, the DCA for the model in the valid ation cohort. The decision curve of the prediction model is composed of an *X*-axis which represents continuum of potential thresholds for NOAF risk and a *Y*-axis which represents the net benefit which is obtained by dividing the net true positives by the sample size. The blue curve "Net benefit: treat all" shows the net benefit if all ACS patients were intervened for NOAF. The red lind "Net benefit: treat none" shows the net benefit if no ACS patients were intervened for NOAF. The black dotted line “prediction model” curve shows the net benefit if it is used to select patients for NOAF intervention.


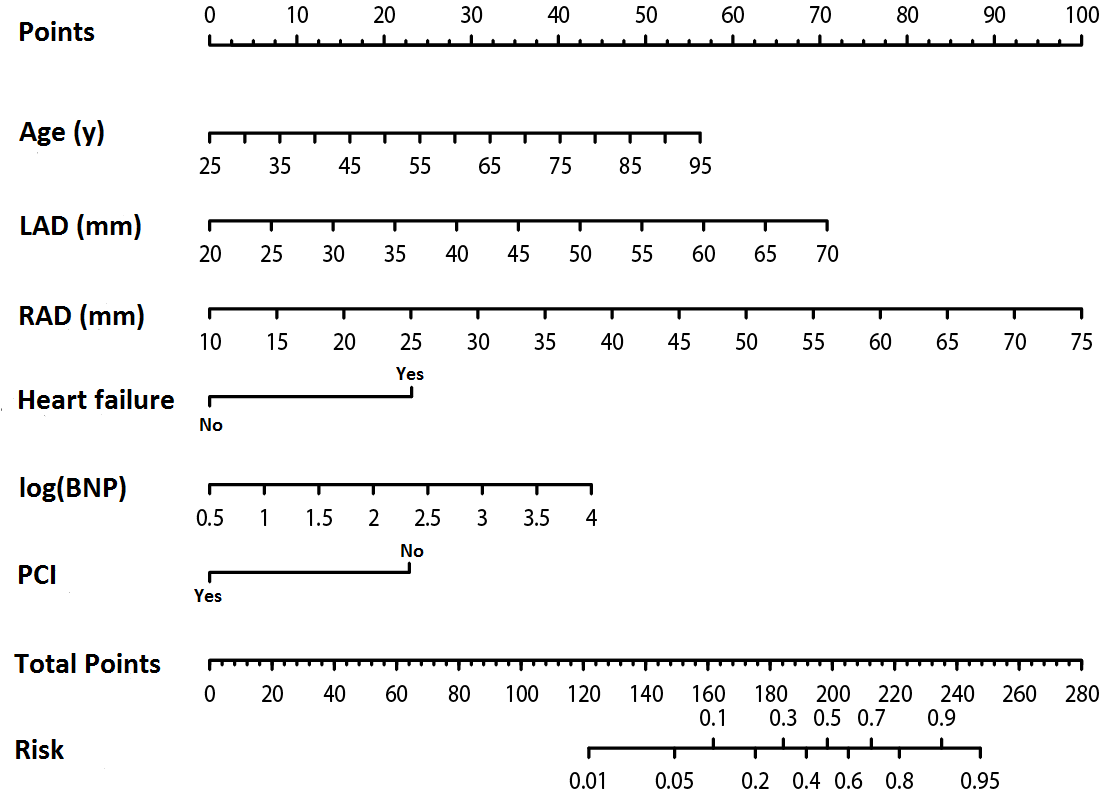


**Supplemental Figure S4.** Nomogram to predict NOAF risk during hospitalization in UA patients. To use the nomogram, an individual patient’s value was located on each variable axis, and a line was drawn upward to determine the number of points received for each variable value. The sum of these numbers was located on the Total Points axis, and a line was drawn downward to the risk axis to determine the risk of NOAF presence. LAD, left atrial diameter; RAD, right atrial diameter; BNP, brain natriuretic peptide; PCI, percutaneous coronary intervention; HR, heart rate.
